# Exploring brain asymmetry in early‐stage Parkinson's disease through functional and structural MRI

**DOI:** 10.1111/cns.14874

**Published:** 2024-07-26

**Authors:** Yujing Liu, Jiaying Yuan, Changlian Tan, Min Wang, Fan Zhou, Chendie Song, Yuqing Tang, Xv Li, Qinru Liu, Qin Shen, Huang Congli, Jun Liu, Sainan Cai, Haiyan Liao

**Affiliations:** ^1^ Department of Radiology, The Second Xiangya Hospital Central South University Changsha China; ^2^ Clinical Research Center for Medical Imaging in Hunan Province Changsha China

**Keywords:** asymmetry voxel‐based morphometry analysis, early‐diagnosis, Hoehn‐Yahr (H‐Y) stages, Parkinson's disease, resting‐state functional MRI, voxel‐mirrored homotopic connectivity (VMHC)

## Abstract

**Objective:**

This study explores the correlation between asymmetrical brain functional activity, gray matter asymmetry, and the severity of early‐stage Parkinson's disease (PD).

**Methods:**

Ninety‐three early‐stage PD patients (ePD, H‐Y stages 1–2.5) were recruited, divided into 47 mild (ePD‐mild, H‐Y stages 1–1.5) and 46 moderate (ePD‐moderate, H‐Y stages 2–2.5) cases, alongside 43 matched healthy controls (HCs). The study employed the Hoehn and Yahr (H‐Y) staging system for disease severity assessment and utilized voxel‐mirrored homotopic connectivity (VMHC) for analyzing brain functional activity asymmetry. Asymmetry voxel‐based morphometry analysis (VBM) was applied to evaluate gray matter asymmetry.

**Results:**

The study found that, relative to HCs, both PD subgroups demonstrated reduced VMHC values in regions including the amygdala, putamen, inferior and middle temporal gyrus, and cerebellum Crus I. The ePD‐moderate group also showed decreased VMHC in additional regions such as the postcentral gyrus, lingual gyrus, and superior frontal gyrus, with notably lower VMHC in the superior frontal gyrus compared to the ePD‐mild group. A negative correlation was observed between the mean VMHC values in the superior frontal gyrus and H‐Y stages, UPDRS, and UPDRS‐III scores. No significant asymmetry in gray matter was detected.

**Conclusions:**

Asymmetrical brain functional activity is a significant characteristic of PD, which exacerbates as the disease severity increases, resembling the dissemination of Lewy bodies across the PD neurological framework. VMHC emerges as a potent tool for characterizing disease severity in early‐stage PD.

## Graphical abstract

This graphical abstract depicts evolving brain functional asymmetry in early‐stage Parkinson's disease (ePD). Comparing ePD mild and moderate groups with controls reveals increasing asymmetry akin to Lewy body spread from lower to higher brain regions. This insight offers a novel perspective on PD neuropathology and a potential progression biomarker.

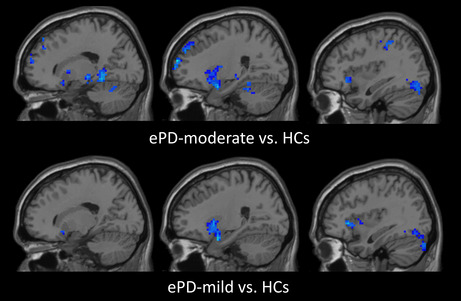

## 1 INTRODUCTION

Parkinson's disease (PD) ranks as the second most common neurodegenerative disorder,^1^ marked by a spectrum of progressive motor and non‐motor symptoms.^2^ Early intervention holds the potential to decelerate PD progression^3^; however, the early stages of the disease often face challenges of misdiagnosis and underdiagnosis, complicating the accurate monitoring of disease progression and the adjustment of treatment strategies.^4^, ^5^ Therefore, exploring the characteristics of early‐stage PD is of significant importance.^6^, ^7^

A defining neuropathological characteristic of PD is the abnormal accumulation of alpha‐synuclein (α‐SN) within neurons, leading to the formation of Lewy bodies.^8^ This process is closely associated with progressive intraneuronal accumulation, ultimately culminating in neurodegeneration. There is strong evidence supporting a prion‐like spreading mechanism of misfolded α‐SN, which is believed to correlate with the stages of PD progression.^9^, ^10^ Given the brain's inherent ipsilateral connectivity, it is speculated that this pathological process may spread asymmetrically, causing unilateral and asymmetric neurodegeneration.^11^ In fact, the asymmetry in clinical severity is a notable feature of PD, where motor symptoms often tend to be more pronounced on one side of body, reflecting that the extent of damage is not consistent across the bilateral cerebral hemispheres.^12^ Additionally, previous studies have identified asymmetric dopaminergic impairment/dysfunction in the substantia nigra and striatum,^13^ as well as asymmetric damage in cortical areas and white matter.^14^, ^15^ Building on these findings, we propose that the asymmetric spread of α‐synuclein within the brain may manifest as asymmetrical damage in brain function and structure.

Voxel‐Mirrored Homotopic Connectivity (VMHC) assesses the symmetry of functional activities of the brain by calculating the correlation coefficients of the activity curves from corresponding voxels.^16^ Researchers utilizing VMHC have discovered significant asymmetries in functional activities across various brain regions in PD.^17^ Further studies suggest that many symptoms of PD may be linked to this interhemispheric functional asymmetry. For example, Li et al. found that the freezing of gait in PD is associated with reduced VMHC in the inferior parietal lobule^18^; Liao et al. observed that depression in PD might arise from asymmetries in the medial frontal gyrus and paracentral lobule^19^; and Zhang et al. noted asymmetrical functional activities in the cortical limbic and nigrostriatal systems in PD with apathy.^20^ However, there has been limited focus on the relationship between the symmetry of interhemispheric functional activity and the severity of PD.

Asymmetry Voxel‐Based Morphometry (VBM) allows for the calculation of the asymmetry index (AI), which quantifies the asymmetry in gray matter volume between the hemispheres.^21^ Although numerous studies using VBM have identified gray matter volume reduction in PD,^22^, ^23^, ^24^ research into the asymmetry of gray matter volume between the cerebral hemispheres in PD remains limited.

This study aims to investigate the relationship between asymmetry in brain functional activity and gray matter volume with the severity of early‐stage PD. Utilizing the Hoehn and Yahr (H‐Y) staging system for disease severity assessment and VMHC analysis for evaluating brain functional activity asymmetry,^16^ alongside asymmetry VBM for assessing gray matter volume asymmetry,^21^ we hypothesize that increased disease severity will correspond with heightened brain structure and function asymmetry.

## 2 MATERIALS AND METHODS

### 2.1 Participants

After obtaining approval from the Medical Ethics Committee of our hospital, we systematically recruited idiopathic PD patients from our Department of Neurology between February 2018 and October 2021, adhering to the Movement Disorder Society (MDS) PD diagnostic criteria.^25^ Age‐, sex‐, and education‐matched normal controls were recruited from the community. After securing informed consent, we collected participants' demographics, Hamilton Depression Rating Scale (HAMD‐17) scores, and Mini‐Mental State Examination (MMSE) scores. For PD patients, additional data on the initial side of motor symptom onset, disease duration, Unified Parkinson's Disease Rating Scale (UPDRS) scores, and Hoehn and Yahr (H‐Y) stages were collected.

The inclusion criteria for this study were: (1) cessation of anti‐PD medications for 12 h (clinical “off” state). (2) sufficient understanding of the purpose and risks of the study, and voluntary participation; (3) clinical diagnosis of PD according to MDS PD criteria, with HY stages at 1–2.5; (4) right‐handedness; (5) head motions <2.5 mm of translation or 2.5 of rotation in any direction during MRI scans. The exclusion criteria included: (1) significant cognitive impairment as assessed by MMSE (scores below 17 for illiterate individuals, below 20 for those with 1–6 years of education, and below 23 for those with 7 or more years of education); (2) presence of mass brain lesions (such as severe head trauma, cerebrovascular disease, epilepsy, or history of neurosurgery); and (3) history of other neurological or psychiatric diseases. This culminated in 93 eligible early‐stage PD patients (H‐Y stages 1–2.5), including 47 in the mild group (H‐Y stages 1–1.5) and 46 in the moderate group (H‐Y stages 2–2.5), alongside 43 matched controls.

### 2.2 Magnetic resonance data acquisition

A Siemens 3.0 T MRI scanner (MAGNETOM Skyra, Germany) was used to collect all MRI images. Participants were prepped with earplugs and foam padding to minimize noise and movement. Instructions were given to remain still, eyes closed, awake, and mind clear during scans. A 3D volumetric magnetization‐prepared rapid gradient‐echo (3D‐MP‐RAGE) sequence was used to acquire three‐dimensional T1‐weighted anatomical images with the following parameters: Number of layers (Sagittal) = 176, Layer thickness = 1.0 mm, TR = 1900.0 ms, TE = 2.01 ms, FA = 9°, FOV = 256 × 256 mm. Rest‐state Functional MRI (r‐fMRI) images were acquired using an EPI sequence with the following parameters: echo time (TE) = 25 ms; repetition time (TR) = 2500 ms; voxel size = 3.75 × 3.75 × 3.5 mm; flip angle (FA) = 90°; field of view (FOV) = 240 × 240 mm^2^, data matrix = 64 × 64; slice gap = 0 mm; slice thickness = 3.5 mm; 39 interleaved slices and 200 volumes.

### 2.3 MRI data preprocessing for voxel‐mirrored homotopic connectivity analysis

First, the data were converted from Digital Imaging and Communications in Medicine (DICOM) format to Neuroimaging Informatics Technology Initiative (NIfTI) format using the dicom2nii toolbox.^26^ Subsequent preprocessing operations were carried out on the Matlab 2021a platform (The MathWorks Inc., Natick, MA, USA), using the DPABI software package, version 6.2 (available at: http://www.restfmri.net/forum/dparsf). The preprocessing steps are as follows: (1) removal of the first 10 time points, retaining the remaining 190 volumes; (2) correction of slice timing differences; (3) head motion correction by the Friston 24‐parameter model^27^; (4) realignment and application of skull stripping for better registration of T1 structural images with functional images; (5) segmentation of T1 images into gray matter, white matter, and cerebrospinal fluid using the New Segmentation + DARTEL algorithm, and “normalization” of the segmented images to a standard template (Montreal Neurological Institute) using DARTEL algorithm; (6) spatial normalization of functional images using the normalization parameters obtained from the previous preprocessing step, and resampling to 3 × 3 × 3 mm; (7) Gaussian smoothing with a 6 mm full‐width at half‐maximum kernel (FWHM); (8) removal of linear trends, and regression of white matter signals, cerebrospinal fluid signals, and Friston 24 head motion parameters as covariates; (9) temporally bandpass filtering (0.01–0.1 Hz).

### 2.4 Voxel‐mirrored homotopic connectivity analysis

Voxel‐mirrored homotopic connectivity analysis is a method used in resting‐state functional magnetic resonance imaging (rs‐fMRI) analysis, which calculates the functional connectivity between symmetric voxels, reflecting the symmetry of functional activity between the brain hemispheres. Lower VMHC values indicate higher asymmetry in functional activity.

VMHC analysis was performed using the DPABI software package, version 6.2 (available at: http://www.restfmri.net/forum/dparsf). The steps involved are as follows: (1) Average all subjects' spatially normalized T1‐weighted imaging (T1WI) images to create an average T1WI; (2) Mirror and flip the average T1WI; (3) Average the average T1WI and its flipped version to generate a symmetric brain template; (4) Non‐linearly register each subject's spatially normalized T1WI to the symmetric brain template to obtain transformation parameters; (5) Apply the transformation parameters to the preprocessed resting‐state fMRI (rs‐fMRI) images; (6) Calculate the correlation coefficient between the time signal curves of each voxel and its corresponding voxel located in the opposite hemisphere; (7) Enhance the normality by performing a Fisher z‐transformation, and the resulting z‐transformed correlation coefficient is the VMHC value.

### 2.5 Asymmetry voxel‐based morphometry analysis

In analyzing the asymmetry of gray matter volume, we utilized the asymmetry VBM method. This method utilized 3D T1WI and was implemented using the VBM8 toolbox (http://dbm.neuro.uni‐jena.de/vbm/download/) within the SPM8 software, referencing prior literature.^21^ The main steps included: (1) Removing the scalp and skull from T1‐weighted images; (2) Registering T1WI to a symmetric tissue probability atlas; (3) Segmenting T1WI into gray matter, white matter, and cerebrospinal fluid; (4) Flipping the gray and white matter images left to right; (5) Using the DARTEL algorithm to create brain templates from the original and flipped gray and white matter images, and registering each subject's original and flipped gray and white matter images to their respective brain templates; (6) Generating grayscale images based on the gray matter volume asymmetry index (AI), retaining only the right hemisphere for further analysis. The calculation formula is AI image = ((i1 − i2)/((i1 + i2) * 0.5)) * i3, where i1 = the original gray matter image after DARTEL registration, i2 = the left–right flipped gray matter image, and i3 = the right hemisphere mask; (7) Applying spatial smoothing to each AI image with an 8 mm FWHM.

Quality control involved visual assessment by two radiologists unaware of subject information. Additionally, explicit masks were created to mitigate noise interference for subsequent statistical analysis.

### 2.6 Statistical analysis of clinical data

Statistical analysis of demographic and clinical data was conducted using the SPSS statistical analysis software (version 27.0; SPSS Inc. Chicago, IL, USA). For continuous variables, we first tested the data for normality and homogeneity of variance using the Shapiro–Wilk test and Levene's test, respectively. The participants' age and education years met the requirements for normal distribution and homogeneity of variance, while other clinical data did not (*p* < 0.05). Accordingly, we selected the *χ* ^2^ test, one‐way analysis of variance (ANOVA), Welch's ANOVA, and Mann–Whitney test for statistical analysis, as appropriate for the data characteristics. See Table 1 for details.

**TABLE 1:** Demographic and clinical characteristics of all participants.

Item	ePD‐moderate	ePD‐mild	HCs	*p* Value	Post hoc (Bonferroni corrected)
Number	46	47	43	–	
Age (years)	58.20 ± 8.47	56.15 ± 10.32	55.14 ± 7.23	0.250^a^	
Sex (M/F)	22/24	30/17	19/24	0.135^b^	
Education (years)	7.35 ± 3.83	7.49 ± 4.19	7.43 ± 3.10	0.983^a^	
MMSE	26.37 ± 2.30	26.66 ± 2.39	27.09 ± 2.62	0.471^c^	
HAMD‐17	7.24 ± 6.46	6.04 ± 6.92	2.02 ± 2.36	<0.001^c^	PD‐mild >HC (*p* < 0.001) PD‐moderate>HC (*p* < 0.001)
H‐Y stages	2.25 ± 0.25	1.19 ± 0.25	–	<0.001^d^	
Side of onset (L/R)	20/26	18/29	–	0.611^b^	
UPDRS	32.91 ± 20.60	20.60 ± 12.77	–	<0.001^d^	
UPDRS‐III	21.61 ± 11.92	11.84 ± 8.67	–	<0.001^d^	
Duration (y)	3.18 ± 4.57	1.60 ± 1.63	–	0.012^d^	

*Note*: Data are presented as mean ± standard deviation. *p*‐value <0.05 was considered statistically significant.

Abbreviations: “–”, not applicable; ePD‐mild, early‐stage Parkinson's disease with the H‐Y stages in 1.0–1.5; ePD‐moderate, early‐stage Parkinson's disease with the HY stages in 2.0–2.5; F, female; HAMD‐17, 17‐item Hamilton Depression Rating Scale; HCs, healthy controls; H‐Y stages, Hoehn‐Yahr clinical rating scale; L, left; M, male; MMSE, Mini‐Mental State Examination; R, right; Side of onset, the initial side of onset of motor symptoms; UPDRS, Unified Parkinson's Disease Rating Scale; UPDRS‐III, motor section of the UPDRS.

^a^ One‐way ANOVA.

^b^ Chi‐square test.

^c^ Welch's ANOVA.

^d^ Mann‐Whitney *U* test.

### 2.7 Statistical analysis of VMHC and AI images

The statistical analyses of VMHC and AI were conducted using the DPABI software package. Initially, a one‐way analysis of covariance (ANCOVA) was performed to determine significant differences in VMHC/AI values among the early PD group, moderate early PD group, and healthy control (HC) group in specific brain regions. Subsequently, these regions were extracted as masks, and a two‐sample post‐hoc *t*‐test was conducted within each mask to detect significant differences between groups. For the statistical analysis of VMHC, age, gender, and education level were included as covariates. For the AI analysis, age, gender, education level, total intracranial volume (TIV), and the side of onset were included as covariates. Gaussian random field correction (GRF) was utilized to correct for multiple comparisons in all analyses (voxel‐level *p* < 0.001, cluster‐level *p* < 0.01) (Chen et al., 2018). Finally, the average VMHC/AI values of brain regions exhibiting significant differences between the PD‐mild group and PD‐moderate group were extracted, and correlations between the average VMHC/AI values and clinical scales were calculated to explore potential associations.

## 3 RESULTS

### 3.1 Demographics and clinical characteristics

The demographic and clinical characteristics of all participants are summarized in Table 1. No significant differences were observed among the groups regarding age, sex, years of education, MMSE scores, and the initial side of onset of motor symptoms (*p* > 0.05). As expected, significant differences were found in H‐Y stages, disease duration, UPDRS scores, and UPDRS‐III scores between the ePD‐mild and ePD‐moderate groups (*p* < 0.05). HAMD scores were higher in both PD subgroups compared to the healthy control group (*p* < 0.05), with no significant difference between the ePD‐mild and ePD‐moderate groups.

### 3.2 Inter‐group differences in VMHC

**FIGURE 1:**
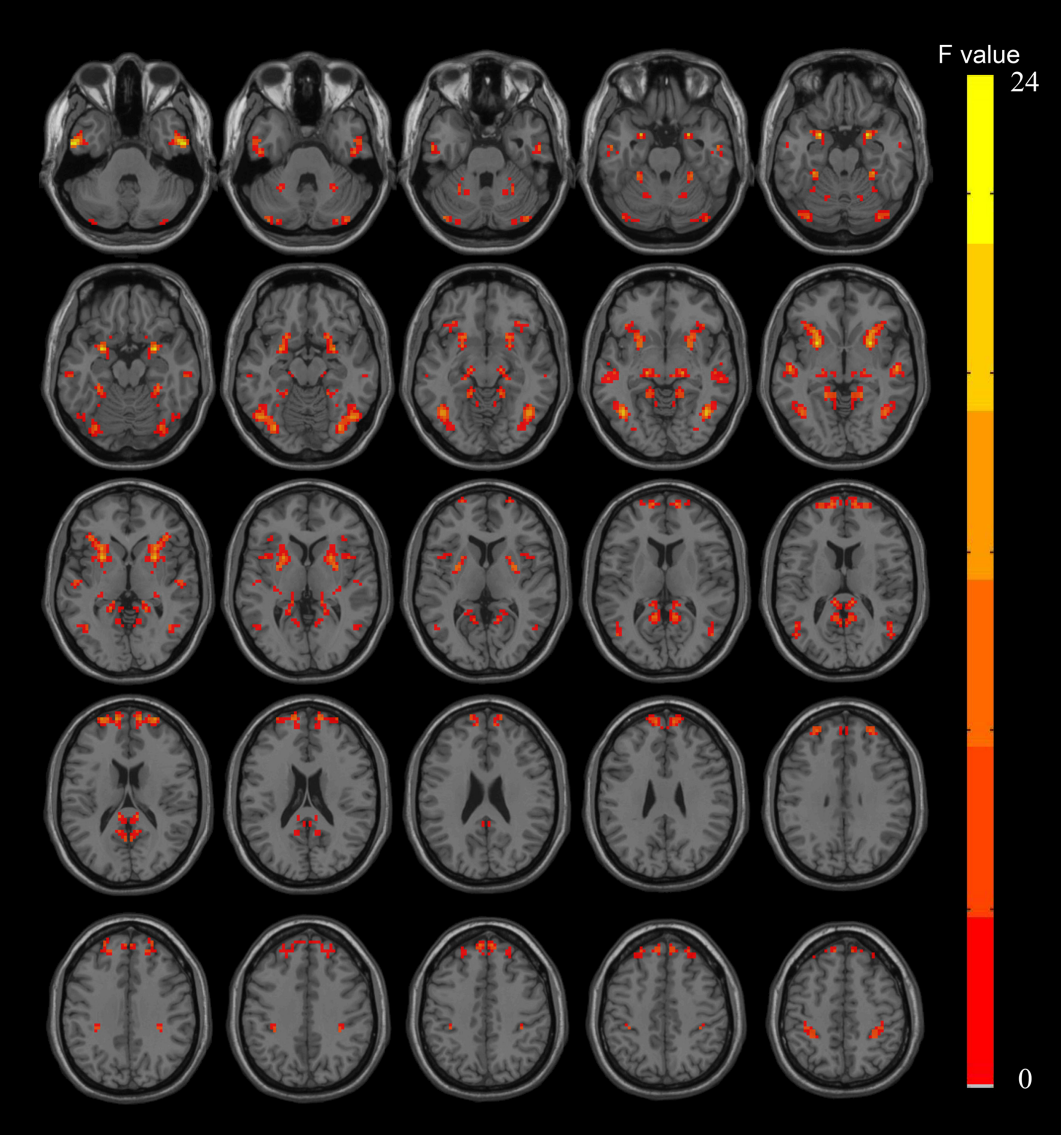
ANCOVA identified brain regions with significant differences in VMHC values among the three groups.

**FIGURE 2:**
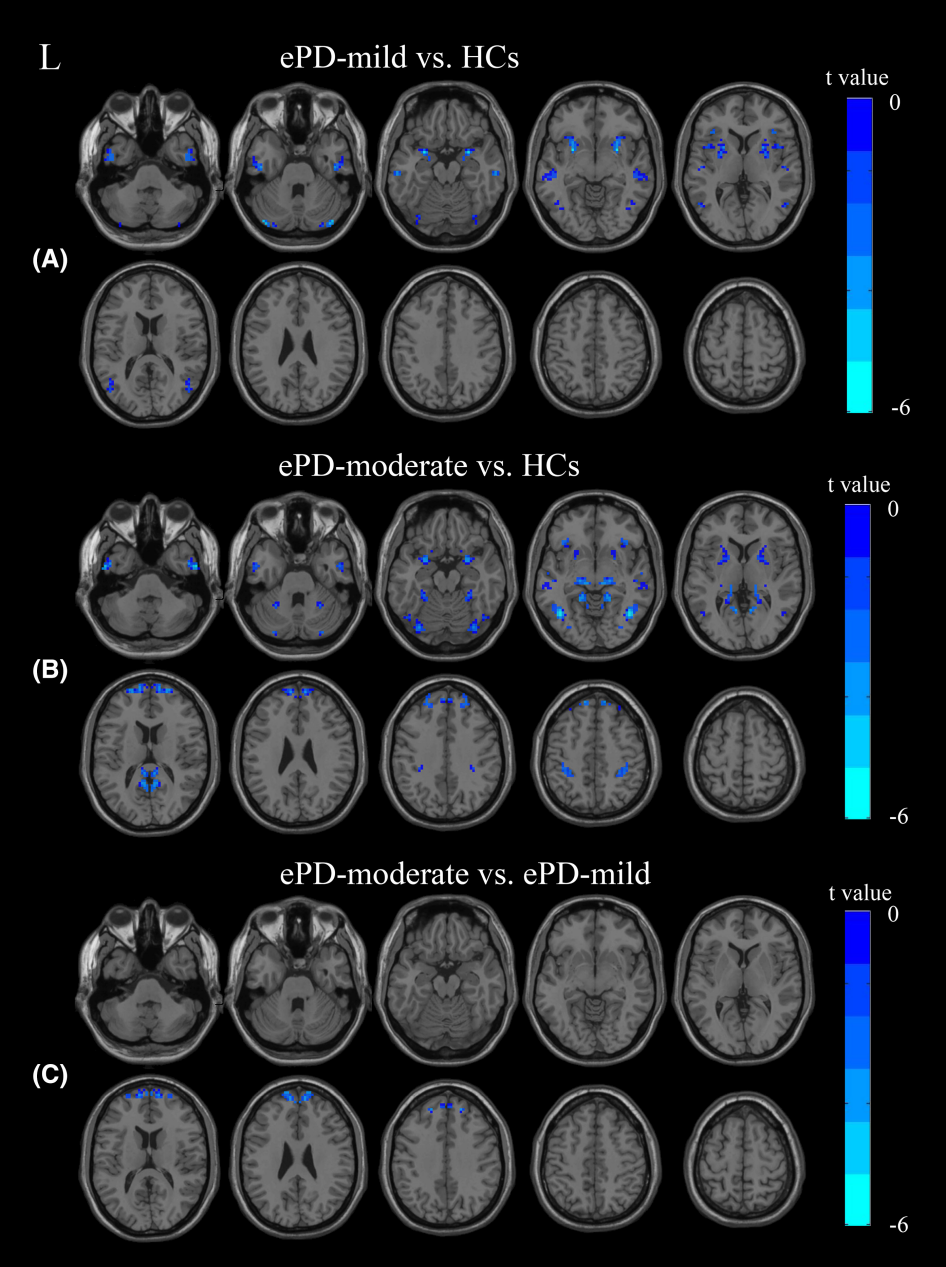
Brain regions showing significant VMHC values differences between each of the two groups in post‐hoc two sample *t*‐test. (A) Comparison between the ePD‐mild and HCs. (B) Comparison between the ePD‐moderate and HCs. (C) Comparison between the ePD‐moderate and ePD‐mild group. ePD‐mild early‐stage Parkinson's disease at H‐Y stages 1.0–1.5, ePD‐moderate early‐stage Parkinson's disease at H‐Y stages in 2.0–2.5, HCs healthy controls. See Table 2 for more detail.

ANCOVA revealed brain regions with significant VMHC value differences among the three groups (Figure 1). These regions were used as masks for subsequent post‐hoc two‐sample *t*‐tests to explore VMHC value differences between pairs of groups. The findings (Figure 2; Table 2) indicate that compared to HCs, the ePD‐mild group exhibited decreased VMHC values in the amygdala, putamen, inferior temporal gyrus, middle temporal gyrus, and cerebellum crus I (Figure 2A; Table 2). The ePD‐moderate group showed reduced VMHC values in these areas plus the lingual gyrus, postcentral gyrus, and superior frontal gyrus compared to HCs (Figure 2B; Table 2). The ePD‐moderate group demonstrated lower VMHC in the superior frontal gyrus than the ePD‐mild group (Figure 2C; Table 2).

**TABLE 2:** Brain regions showing significant VMHC values differences between each two groups.

Groups	Brain region (AAL atlas)	Cluster size	Peak MNI coordinates (*x y z*)			*t*‐value
ePD‐mild vs. HCs						
Cluster1	Amygdala	198	±27	3	−18	−5.69
	Putamen					
Cluster2	Cerebelum_Crus1	116	±39	−84	−30	−5.07
	Temporal_Inf					
Cluster3	Temporal_Inf	73	±45	−15	−30	−4.61
Cluster4	Temporal_Mid	55	±54	−18	−3	−4.87
Cluster5	Temporal_Mid	27	±51	−63	6	−4.48
ePD‐moderate vs. HCs						
Cluster1	Lingual	222	18	−42	9	−5.34
Cluster2	Temporal_Inf	210	42	−60	−6	−5.92
	Cerebelum_Crus1					
Cluster3	Frontal_Sup	190	±24	63	18	−5.60
Cluster4	Amygdala	155	±24	3	−21	−5.35
	Putamen					
Cluster5	Temporal_Inf	61	±57	−6	−36	−5.72
Cluster6	Postcentral	45	±33	−36	51	−5.17
Cluster7	Temporal_Mid	23	±54	−15	−3	−4.81
ePD‐moderate vs. ePD‐mild						
Cluster1	Frontal_Sup_Medial	109	±12	63	21	−5.190

*Note*: Results were corrected by GRF (voxel‐level *p* < 0.001, cluster‐level *p* < 0.01). Negative *t*‐value indicates that the VMHC values of the former group are lower than the latter group.

Abbreviations: AAL atlas, Anatomical Automatic Labeling atlas; ePD‐mild, early‐stage Parkinson's disease with the H‐Y stages in 1.0–1.5; ePD‐moderate, early‐stage Parkinson's disease with the H‐Y stages in 2.0–2.5; HCs, healthy controls; MNI, Montreal Neurological Institute.

### 3.3 Inter‐group differences in AI

No statistically significant differences were found in AI images among the three groups.

### 3.4 Correlation analysis

**FIGURE 3:**
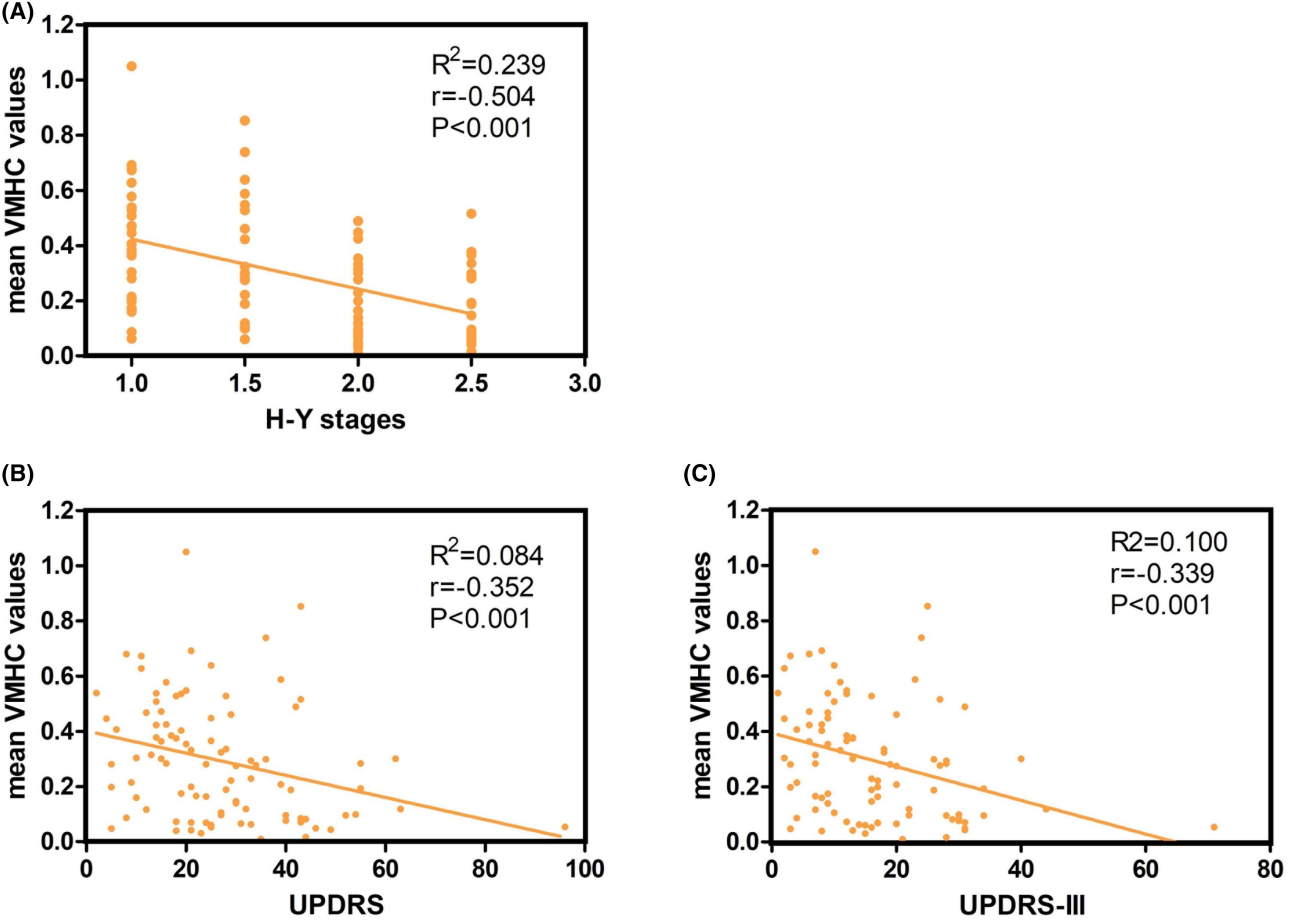
Correlation analysis between the average VMHC values of the medial superior frontal gyrus in PD patients and clinical scales.(A) The VMHC value of superior frontal gyrus was negatively correlated with Hoehn and Yahr Stages (H‐Y stages). (B) The VMHC value of superior frontal gyrus was negatively correlated with Unified Parkinson's Disease Rating Scale scores (UPDRS). (C) The VMHC value of superior frontal gyrus was negatively correlated with motor section of Unified Parkinson's Disease Rating Scale scores (UPDRS‐III).

Following the identification of inter‐group VMHC differences, we analyzed the mean VMHC values of the superior frontal gyrus across all PD patients. Correlation analyses between these VMHC values and disease duration, H‐Y staging, UPDRS, UPDRS‐III, MMSE, and HAMD‐17 scores were conducted. The results (Figure 3) show significant negative correlations between the superior frontal gyrus VMHC values and UPDRS, UPDRS‐III scores, and H‐Y stages. However, no significant correlations were found with disease duration, MMSE scores, or HAMD‐17 scores (Spearman correlation, Bonferroni corrected, *p* < 0.01/6 = 0.001).

## 4 DISCUSSION

Our research into early‐stage PD has revealed significant asymmetry in cross‐hemispheric functional activity across certain brain regions, which appears to progressively spread in a pattern reminiscent of Lewy body propagation as described by Braak.^28^ Initially, our findings show that the ePD‐mild group exhibited reduced VMHC values in the amygdala, caudate nucleus, inferior temporal gyrus, middle temporal gyrus, and cerebellar Crus I compared to healthy controls (HCs). Furthermore, the ePD‐moderate group displayed additional reductions in VMHC values in the posterior central gyrus, lingual gyrus, and superior frontal gyrus compared to HCs, beyond the abnormalities observed in the ePD‐mild group. Notably, the ePD‐moderate group showed a decrease in VMHC values in the superior frontal gyrus relative to the ePD‐mild group, correlating negatively with H‐Y stage. However, no significant asymmetry was detected in the gray matter structure of early‐stage PD patients.

The symmetrical functional activity between corresponding regions of the two cerebral hemispheres highlights the importance of synchronized activity for optimal brain function.^16^, ^29^ Luo et al. discovered that PD exhibits significantly reduced VMHC in the caudate and cortical areas.^17^ These regions are closely related to sensory processing and motor control and play a crucial role in PD. Our research findings are largely consistent with theirs, and we further noted that VMHC abnormalities in these areas vary among PD patients with different severity levels. The abnormalities in VMHC may worsen as the disease severity increases.

### 4.1 Interhemispheric functional asymmetry begins in the limbic system and basal ganglia

Our research indicates that the early‐stage PD mild group exhibited impaired interhemispheric synchrony in limbic system (amygdala, temporal gyrus) and basal ganglia (putamen) compared to healthy controls, areas typically implicated in Braak's pathological stages 3–4. The limbic system plays a crucial role in emotional perception, processing, and regulation. Abnormalities in function or structure of the limbic system are often associated with depression symptoms in PD,^30^, ^31^ which commonly emerge in the early stages of PD,^2^ aligning with our observations of desynchronized interhemispheric functional connectivity of bilateral limbic system in ePD mild group. The putamen is a vital component of the dopaminergic pathway within the nigrostriatal system, and dysfunction in this pathway is a well‐known mechanism underlying the motor symptoms of PD. Thus, the observed discoordination in bilateral putamen may be associated with the emergence of motor symptoms in the early‐stage PD mild group. Since the early‐stage PD mild group only exhibits motor symptoms on one side of the body, we hypothesize that the observed functional asymmetry in the bilateral caudate may be related to more severe impairment in the function of one caudate. Additionally, research has shown that a reduction in the VMHC of the caudate is closely related to the apathy symptoms of PD.^20^ Apathy typically manifests in the early stages of the disease, which is consistent with our research findings.

### 4.2 As the severity increases, interhemispheric functional asymmetry spreads to more neocortex areas

In addition to the existing abnormalities observed in the mild group, the early‐stage PD moderate group displayed impaired interhemispheric synchrony in postcentral gyrus, lingual gyrus, and superior frontal gyrus. These regions are associated with high order cognitive and motor functions. Lewy bodies typically spread to these regions in Braak pathological stages 5–6. The postcentral gyrus serves as the primary region for somatosensory cortex, directly synapsing with the motor cortex and pre‐motor cortex in the frontal lobe, contributing to the integration of sensory and motor functions.^32^ Dysfunction in the postcentral gyrus may contribute to the overlay of abnormal sensory processing on motor function, ultimately leading to the motor symptoms of PD.^33^ Additionally, research has shown that reduced VMHC value of the postcentral gyrus is associated with freezing of gait in PD.^18^ This symptom typically appears in the more severe stages of motor symptoms, which aligns with our research findings. The lingual gyrus, located in the occipital lobe, is a part of the secondary visual cortex and is primarily involved in visual spatial processing.^34^ Increasing evidence suggests that abnormalities in the visual network are closely associated with cognitive impairment in PD, especially in the domains of visual spatial processing and memory.^35^, ^36^ The superior frontal gyrus, a component of the prefrontal cortex (PFC), known for its association with cognitive functions like attention, working memory, and executive control.^37^ Several studies have observed structural or functional abnormalities of the PFC in PD with cognitive impairments.^38^, ^39^ Moreover, the PFC has direct connections to the motor cortex and plays a compensatory role in non‐automatic movement execution and postural control.^40^ Therefore, functional abnormalities in the prefrontal cortex of PD patients are also associated with gait disturbances and increased risk of falling.^41^ Thus, the observed desynchronization between bilateral PFC‐related areas may account for the postural reflex abnormalities of PD.

In summary, the impaired interhemispheric synchrony in the ePD‐moderate group extends to more neocortical regions associated with cognitive and motor functions, aligning with the increasing severity of PD.

### 4.3 Interhemispheric functional asymmetry of PFC indicates more severe PD conditions

Compared to the ePD‐mild group, a characteristic feature in the ePD‐moderate group is the impaired interhemispheric synchrony in the superior frontal gyrus, which is a part of the PFC. As noted earlier, the PFC is associated with non‐automatic execution of movement and posture control. Correlation analysis reveals a negative correlation between the mean VMHC values of the bilateral superior frontal gyrus and the HY stages, the Unified Parkinson's Disease Rating Scale (UPDRS), and UPDRS‐III scores, further underscoring the role of the PFC in reflecting the severity of motor symptoms in PD.

In summary, our study indicates that decreased interhemispheric synchrony in the superior frontal gyrus may serve as an approach to differentiate between varying severity levels in early‐stage PD. Combined with our previous finding that the regional homogeneity (ReHo) values of the orbitofrontal gyrus can distinguish between mild and moderate early‐stage PD patients,^7^ we believe that the unique significance of PFC in the progression of early‐stage PD should be given more attention in research.

### 4.4 Interhemispheric gray matter volume asymmetry is not significant in early‐stage PD

Our study did not find significant asymmetry in interhemispheric gray matter volume between PD patients and HCs, consistent with previous research findings.^20^ We hypothesize this may be because, in the early stages of PD, brain function is more susceptible to disruption than brain structure. Additionally, current VBM analysis may not capture some subtle or complex changes in brain structure. Therefore, future research should develop surface‐based morphology methods for analyzing structural asymmetry, providing a more comprehensive analysis of early‐stage PD from aspects such as interhemispheric cortical thickness, sulcal depth, gyrification index, and cortical complexity. From another perspective, the symmetry of gray matter volume also supports our findings on functional asymmetry, indicating that these results of functional asymmetry are not illusions caused by structural asymmetry, thus validating the reliability of our findings on functional asymmetry.

### 4.5 Limitation

Our study has several limitations. First, we conducted a cross‐sectional study comparing different H‐Y stages of early‐stage PD in terms of brain asymmetry, which cannot establish causality. Further longitudinal studies are needed to confirm our findings. Second, although participants were matched for age, gender, and education level to control for confounding factors, the heterogeneity of PD may still introduce unaccounted confounding variables. Third, the PD patients in this study were not in a drug‐naive state. Despite a 12‐h withdrawal period to mitigate the pharmacological effects of anti‐Parkinsonian medication on neural activity, long‐term medication treatment may introduce potential confounding effects that may not be entirely avoided. Fourth, the methodological limitations of VBM necessitate further exploration of gray matter structure through surface‐based analysis for a comprehensive understanding.

## 5 CONCLUSION

We discovered that the progressively spreading asymmetrical brain functional activity is a crucial feature in early‐stage PD patients, resembling the propagation of Lewy bodies and the exacerbation of symptoms. These findings may aid in the early diagnosis of PD and its progression monitoring, offering new insights into the cerebral functional abnormalities and pathological changes in PD.

## CONFLICT OF INTEREST STATEMENT

The authors declare that the research was conducted in the absence of any commercial or financial relationships that could be construed as a potential conflict of interest.

## Data Availability

The data that support the findings of this study are available from the corresponding author upon reasonable request.
